# Micro-RNAs as Potential Predictors of Response to Breast Cancer Systemic Therapy: Future Clinical Implications

**DOI:** 10.3390/ijms18061182

**Published:** 2017-06-02

**Authors:** Alma D. Campos-Parra, Gerardo Cuamani Mitznahuatl, Abraham Pedroza-Torres, Rafael Vázquez Romo, Fany Iris Porras Reyes, Eduardo López-Urrutia, Carlos Pérez-Plasencia

**Affiliations:** 1 Laboratorio de Genómica, Instituto Nacional de Cancerología (INCan), Av. San Fernando 22, Col. Sección XVI, C.P. 14080 Tlalpan, Ciudad de México, Mexico; cuamanigm@hotmail.com (C.M.G.); abraneet@gmail.com (P.-T.A.); 2 CATEDRA-CONACyT, Av. De los Insurgente Sur 1582, Col. Crédito Constructor., C.P. 03940 Benito Juárez, Ciudad de México, Mexico; 3 Departamento de Cirugia de Tumores mamarios, Instituto Nacional de Cancerología (INCan), Av. San Fernando 22, Col. Sección XVI, C.P. 14080 Tlalpan, Ciudad de México, Mexico; vrrafa@yahoo.com.mx; 4 Servicio de Anatomia Patologica, Instituto Nacional de Cancerología (INCan), Av. San Fernando 22, Col. Sección XVI, C.P. 14080 Tlalpan, Ciudad de México, Mexico; fany.porras@gmail.com; 5 Unidad de Biomedicina, FES-IZTACALA, Universidad Nacional Autónoma de Mexico (UNAM), Av. De Los Barrios 1, Col. Los Reyes Iztacala, C.P. 54090 Tlalnepantla, México, Mexico; e.urrutia@me.com (L.-U.E.); carlos.pplas@gmail.com (P.-P.C.)

**Keywords:** breast cancer, miRNAs, response to systemic therapy

## Abstract

Despite advances in diagnosis and new treatments such as targeted therapies, breast cancer (BC) is still the most prevalent tumor in women worldwide and the leading cause of death. The principal obstacle for successful BC treatment is the acquired or de novo resistance of the tumors to the systemic therapy (chemotherapy, endocrine, and targeted therapies) that patients receive. In the era of personalized treatment, several studies have focused on the search for biomarkers capable of predicting the response to this therapy; microRNAs (miRNAs) stand out among these markers due to their broad spectrum or potential clinical applications. miRNAs are conserved small non-coding RNAs that act as negative regulators of gene expression playing an important role in several cellular processes, such as cell proliferation, autophagy, genomic stability, and apoptosis. We reviewed recent data that describe the role of miRNAs as potential predictors of response to systemic treatments in BC. Furthermore, upon analyzing the collected published information, we noticed that the overexpression of miR-155, miR-222, miR-125b, and miR-21 predicts the resistance to the most common systemic treatments; nonetheless, the function of these particular miRNAs must be carefully studied and further analyses are still necessary to increase knowledge about their role and future potential clinical uses in BC.

## 1. Background

Breast cancer (BC) is the most frequently diagnosed cancer among women worldwide. Over 1 million new cases are diagnosed every year, although early detection and the availability of adjuvant treatments have decreased its mortality [1]. BC is a complex and heterogeneous disease that comprises several molecular subtypes with a dissimilar cellular origin, etiology, response to treatment, and clinical outcome [2]. Current therapy approaches for BC are roughly customized in response to a handful of molecular profiles (luminal, basal-like, and human epidermal growth factor receptor-2 positive) and consists of chemotherapy, endocrine therapy, selective estrogen receptor modulators, and targeted therapies [3].

Different treatment options constitute a considerable advance that has resulted in improved outcomes; however, some types of the disease that comprise a substantial number of patients exhibit tumor progression. The scientific community strives to overcome this challenge and, so far, has described several altered mechanisms that lead to resistance to treatment. These mechanisms include, but are not limited to, intracellular drug depletion via transporters and enzymes, DNA repair pathways, target mutations, resistance to apoptosis, and, recently, the function of microRNAs (miRNAs) [4].

miRNAs are small non-coding ~22 nucleotide-long RNAs that regulate gene expression. Most of them are encoded in RNA polymerase II-associated transcription units that produce a primary miRNA transcript known as pri-miRNA, which, after nuclear cleavage by DROSHA (Double-Stranded RNA-Specific Endoribonuclease) and its cofactor DGCR8 (DiGeorge Syndrome Critical Region Gene 8), gives rise to a 60–70 nucleotide long hairpin structure named pre-miRNA [5]. This pre-miRNA is then exported to the cytoplasm by exportin5 in association with RAN activated GTPase (RAN-GTP), where it is processed by DICER (Double-Stranted RNA-Specific Endoribonuclease) to produce a ~22 base-pair duplex with a 2-nucleotide overhang in its 3′ ends [6]. Subsequently, the two strands of this short dsRNA are separated by RNA helicase and one of them is integrated into the RISC (RNA induced silencing complex), which includes AGO (argonaute) proteins, and the other strand is discarded from the complex [7]. Finally, the complete RISC (RNA-induced silencing complex) binds the target mRNA through miRNA-mRNA base pairing, which leads to protein downregulation [8].

Therefore, the balance between mRNA and miRNAs ensures correct protein expression by keeping mRNA abundance under control. When dysregulated, miRNA expression can be oncogenic by suppressing tumor suppressor genes—these miRNAs are labeled oncomirs; or tumor suppressors, when downregulating oncogenic genes [9].

miRNA dysregulation in BC was first reported in 2005; thenceforth, a plethora of studies have reported the aberrant expression of several miRNAs, both oncomirs and tumor-suppressors. For instance, miR-21 and miR-155 are known oncomirs that disrupt apoptosis and TGF-β signaling, while miR-206 and miR-175p are considered tumor suppressors [10].

However, identifying the dysregulation of miRNAs in cancer onset and development is half of the story, as recent evidence shows that miRNAs are also involved in the process of the response to currently employed treatments with anthracyclines, 5-fluorororacil, cyclophosphamide, carboplatine, taxanes, endocrine, and targeted therapies [11]. In fact, miRNAs appear to perform substantial roles in the observed variations in drug sensitivity from one patient to another, so they are gaining importance as predictors of resistance towards a given treatment.

A considerable amount of research is being conducted to identify miRNAs that take part in these resistance mechanisms; so, in this review, we focus on the description of several independent studies that have evaluated the role of the miRNAs as potential predictors of the response to different treatments in this neoplasia. We hope that this condensed view of available information helps to drive efforts towards therapy individualization. The reports described below are further summarized in Table 1 and Table 2, intended as quick references to the miRNAs that constitute potential biomarkers of resistance and sensitivity to systemic therapy, respectively.

## 2. Main Text

### 2.1. miRNAs in Taxane Resistance (Paclitaxel, Docetaxel)

Taxanes bind the tubulin microtubules and inhibit their depolymerization, affecting mitosis in the G1 or M phase, and are therefore cytotoxic; besides, there is evidence that their antineoplastic activity also comprises interference with biological processes such as apoptosis, angiogenesis, invasiveness, cell motility, and metalloproteinase production [12]. Taxanes—paclitaxel and docetaxel—are considered core drugs in metastatic BC treatment and exhibit different clinical activity, having shown clinical benefits as adjuvants and neoadjuvants, a setting where docetaxel has been more active than paclitaxel [13,14]. Nonetheless, a meta-analysis that compared paclitaxel and docetaxel regimens in metastatic BC demonstrated that both taxanes have a similar effectiveness in terms of overall survival, progression-free survival, and the overall response rate, but paclitaxel was associated with less toxicity and better tolerability. In this regard, and due to the heterogeneity of different trials, more studies are necessary in order to identify patients who will gain the most probable advantage from the appropriate taxane regimen [15]. On the other hand, when two different combined schedules—docetaxel plus gemcitabine versus paclitaxel plus gemcitabine—were compared, both of them showed a similar time to progression in metastatic BC patients (85.6% vs. 87.0% of patients progressing, respectively) [16]. In contrast, a meta-analysis of phase III trials found that combination chemotherapy regiments showed a better time to progression, but not overall survival, in metastatic BC patients [17]. Similarly, it was noticed that a combined schedule, for instance, taxanes (paclitaxel or docetaxel) plus bevacizumab, displayed a higher progression-free survival versus taxanes alone (10 vs. 13.3 months; *p* = 0.474) as first line chemotherapy in metastatic BC [18]. Nonetheless, in spite of several schedule options, patients frequently relapse after chemotherapy treatment as a result of the acquired resistance of the tumor cells or by signals drivens by the microenvironment in order to enhance the cancer stem cells which are resistant to different treatments [19]. A number of miRNAs have been associated to this negative outcome, as summarized below.

### 2.2. Paclitaxel

Resistance to paclitaxel treatment involves the deregulation of several miRNAs. Some of them are overexpressed, such as Lin28 miRNA, a marker of cancer stem cells, whose overexpression was closely associated with the resistance to paclitaxel and was dramatically increased in tumor tissues after neoadjuvant chemotherapy or in local relapse or metastatic BC tissues. Likewise, in BC cells, it was observed that the overexpression of Lin28 miRNA induced p21 and Rb expression and the inhibition of let-7a miRNA levels. In consequence, Lin28 confers certain “stemness” to cancer cells, in order to get the cancer stem cell properties to avoid chemotherapy; on the other hand, Lin28 blocks the processing of let-7a, a tumor suppressor miRNA [20]. Employing an miRNA array, Zhou and colleagues identified that miR-125b, miR-221, miR-222, and miR-923 are upregulated in paclitaxel-resistant BC cells, and found that miR-125b caused a marked inhibition of taxol-induced cytotoxicity and apoptosis through the suppression of Bak1 (pro-apoptotic Bcl2 antagonist killer 1) expression [21]. The role of miR-125b is particularly interesting: it was found to be upregulated in cisplatin-resistant ovarian cells and, in contrast, downregulated in paclitaxel-resistant ovarian cells [21]. This difference shows that the association between miRNA expression and resistance to therapy might not be as straightforward as some studies show, because a single miRNA can play opposite roles in the resistance to different drugs or in different cell types. Therefore, further analysis is necessary to attain the sought after goal of tailored cancer treatments.

Another miRNA involved in paclitaxel-resistance is miR-520h, whose overexpression was associated with a poor prognosis and lymph node metastasis in human BC patients. It’s essential role as a DAPK2 (Death-Associated kinase 2) repressor was identified in cell lines. Interestingly, restoring DAPK2 abolished miR-520h-promoted drug resistance, due to DAPK2 modulation of caspase-dependent apoptosis, which suggested that miR-520h is not only an independent prognosis factor, but also a potential functional target [22].

Likewise, it was demonstrated in an in vitro study that miR-451 influences the sensitivity to neo-adjuvant chemotherapy through the regulation of apoptosis. The overexpression of miR-451 negatively regulates Bcl-2 (Bcl-lymphoma 2) mRNA and protein expression, which increases caspase 3 expression and accelerates apoptosis; hence, this miRNA might stimulate the resistance phenotype of the paclitaxel-resistant BC cell lines [23]. miR-100 sensitizes BC cells to paclitaxel through cell proliferation and survival inhibition by targeting mTOR, as per a set of experiments performed in luminal A, BC cells. Interestingly, in BC patients it was noticed that this miRNA was downregulated in the luminal A subtype, which is associated with a poor prognosis in BC patients treated with chemotherapy, due to the fact luminal A subtype usually responds to hormonal therapies but not to chemotherapies such as paclitaxel [24].

Moreover, it was observed that miR-18a overexpression reduced DICER expression levels and enhanced autophagy via the inhibition of the mTOR signaling pathway, increasing paclitaxel-resistance in triple negative BC cells; the authors point to autophagy inhibition as a novel strategy to improve chemotherapy efficiency [25].

On the other hand, paclitaxel resistance mediated by the downregulation of mRNAs has also been reported, such as let-7a—an miRNA that targets caspase 3 and produces an increase in the resistance to apoptosis induced by paclitaxel, according to an in vitro study that assessed A431 (squamous carcinoma cells) and HepG2 (liver hepatocellular carcinoma) cells [26]. Furthermore, it was reported that let-7f has a pronounced reduction in MCF-7 cells treated with paclitaxel, and the downregulation of let-7 correlated with upregulation of TSP-1 (thrombospondin-1), a validated target of let-7f. Thus, let-7f reduction contributed to TSP-1 upregulation in BC, but did not affect the proliferation and apoptosis of MCF-7 cells [27].

In triple-negative BC, it was noticed that miR-101 was strongly decreased in tissues and cell lines. Its ectopic overexpression inhibited growth and apoptosis in vitro and suppressed tumorigenesis in vivo. The authors demonstrated that MCL-1 (myeloid cell leukemia) was its target, as there was a negative correlation between the level expression of miR-101 and MCL-1 in triple negative BC tissues. Likewise, the downregulation of MCL1 enhanced the expression of cleaved caspase-3 and PARP (Poli ADP ribose polymerase) [28].

### 2.3. Docetaxel

Docetaxel is a semisynthetic analogue of paclitaxel that was synthesized from a precursor extracted from the needles of the European yew [29]. As paclitaxel, docetaxel acts by inhibiting mitotic activity due to the suppression of microtubule depolymerization [29]; whilst also eliciting chemoresistance due to the differential expression of miRNAs. Conversely, clinical trials suggest that docetaxel is less toxic than paclitaxel [14,15].

For example, a study in vitro demonstrated that the overexpression of miR-141 and miR-129-3p was associated with the resistance to docetaxel due to the direct inhibition of EIF4E (eukaryotic translation initiation factor 4E; a molecule associated with apoptosis) and CP110 (centriolar coiled-coil protein 110; which plays an essential role in centrosome duplication), respectively. Therefore, the downregulation of miR-141 leads to an apoptosis-inducing effect through the upregulation of EIF4E. Likewise, the downregulation of CP110 affected apoptosis [30,31].

An upregulated miRNA associated with docetaxel-resistance is miR-3646, through the suppression of GSK-3β expression and the consequent activation of the GSK-3β/β-catenin signaling pathway, which plays important roles in cell proliferation, differentiation, tumorigenesis, and as a chemoresistant of cancer. These results were consistently found in MDA-MB-231 and MCF-7 BC cell lines [32].

Similarly, using docetaxel-resistant MCF-7 and MDAMB-231 cell lines, Hu et al. reported the overexpression of miR-452 and miR-663, respectively. miRNA-452 expression was inversely associated to that of APC4 (anaphase promoting complex subunit 4), while the overexpression of miR-663 downregulated HSPG2 (heparin sulfate proteoglycan 2) and induced chemoresistance; notably, the miR-663 genomic region was hypomethylated in resistant cells [33,34].

In a broader study, through a microarray analysis of MCF-7 and MDA-MB-231 BC cell lines, Kaslt et al. reported miRNA deregulation associated with docetaxel resistance: overexpression of miR-34a and miR-141, and underexpression of miR-7, miR-16, miR-30a, miR-125a-5p, and miR-126. The authors focused on miR-34a since it was significantly upregulated. This miRNA targets BCL-2 and CCND1 (cyclin D1), which are both necessary for signaling pathways or drug-induced apoptosis [35].

A number of downregulated miRNAs play a role in docetaxel resistance. Based on miRNA array data, it was reported that miR-139-5p was significantly downregulated in BC compared with adjacent normal tissue (*n* = 35 BC patients). Evaluating its function in vitro, when restoring its expression artificially with an miR-139-5p mimic, it was observed that it significantly inhibited BC cell growth and, in consequence, inhibited docetaxel-resistance through the induction of apoptosis by targeting Notch1 [36]. Besides, it was reported that miR-205 increases the sensitivity of MCF-7 and MDA-231 cells to docetaxel by inhibiting cell proliferation and clonogenic potential [37].

Similarly, miR-125a was downregulated in docetaxel-resistant BC cells; so its overexpression potentiates docetaxel-sensitivity. This miRNA directly targeted 3’-untranslated regions of *BRCA1*, (Breast Cancer gene) and the suppression of BRCA1 expression improved docetaxel-resistance in BC cells. Likewise, it was noticed that miR-125a was downregulated in HER2-amplified and HER2-overexpressing metastatic BC patients’ specimens (*n* = 32). These findings provide a novel strategy to increase the sensitivity to this drug in BC patients through miR125a overexpression in order to potentiate docetaxel-sensitivity [38].

Combined chemotherapy using docetaxel plus adriamycin is commonly used in the treatment of BC, especially recurrent or metastatic disease; however, the risk of developing a resistance of them remains latent. In this regard, it was reported that, in BC cell lines resistant to docetaxel plus adriamycin, miR-222 and miR-29a were overexpressed. Likewise, it was noticed that miR-222 and miR-29 inhibitors changed the drug-resistance and restored the sensitivity to these drugs through targeting PTEN (phosphatase and tensin homolog), activating the Akt/mTOR pathway (Akt Serine/Threonine Kinase 1/Mechanistic Target of Rapamycin) [39].

A very interesting study indicated that exosomes act as vehicles for the exchange of genetic information between heterogeneous populations of tumor cells and that they can horizontally transfer drug resistance to target cells. Cheng et al. identified the most abundant miRNAs in exosomes derived from docetaxel-resistant cells: miR-1246, miR-23a, miR-1469, miR-38, miR-1915, and let-7b, among others. These findings add yet another layer of complexity to the chemoresistance phenomenon: not only are cells prone to developing resistance upon expositions to specific drugs, they are possibly as likely to spread/acquire it through exosomes [40].

### 2.4. miRNAs in Endocrine Therapy

The specific target of endocrine therapy is the ERα (Estrogen Receptor α). It is over-expressed in about 70% of breast tumors and has been directly linked to the initiation and progression of mammary carcinogenesis [41]. The ER family consists of two nuclear receptors, ERα and ERβ, which are encoded by the *ESR1* and *ESR2* genes, located on chromosomes 6 and 14, respectively. Although both receptors share a similar structure (six structural domains) they are known to have opposite functions: ERα stimulates cell progression and survival, while ERβ acts as a cell proliferation inhibitor [42,43,44].

Endocrine therapy—prescribed for ER-positive BC patients—aims to inhibit the ERα signaling pathway and includes: (A) Estrogen inhibitors, better known as aromatase inhibitors (AI) such as anastrozole, letrozole (non-steroidal origin), and exemestane (steroidal origin), which prevent the synthesis of estrogens through the aromatization of androgens such as testosterone; these have become the first-line endocrine treatment of choice for postmenopausal ER positive BC patients [45]; (B) Selective estrogen receptor downregulators (SERDs), such as fulvestrant, which induce destabilization and subsequent ER degradation; they are used as therapy for patients with metastasic BC after they have developed resistance to aromatase inhibitors and tamoxifen [46,47]; (C) Selective ER modulators (SERMs), such as tamoxifen, which bind directly to the ER, competing with estrogens, and thus inhibit the transcription of genes that respond to ER activity [48].

Despite the demonstrated effect of these drugs, de novo or acquired resistance still constitutes a major problem for their complete success in clinical practice. Several mechanisms of resistance to endocrine therapy have been described, including the loss of ERs, deregulation of PI3K, MAPK, and mTOR signaling pathways, aberrant tamoxifen metabolism, and the deregulation of miRNAs [49,50]. We hereafter describe recent reports that identify miRNAs associated with resistance to aromatase inhibitors and tamoxifen.

Most of these miRNAs have been identified by microarray analysis in BC cell lines that resemble the heterogeneous phenotype and behavior of resistant cells present in the tumor of patients who relapse [51,52], which means that validation in actual patient cohorts is still ahead.

### 2.5. Aromatase Inhibitors

Bacci et al. aimed to identify relevant miRNAs as response predictors to aromatase inhibitors and as putative therapeutic targets on ER positive BC. They used AI (letrozole and anastrozole)-resistant cellular models and identified the deregulated expression of 33 miRNAs in letrozole-resistant BC cells and 18 miRNAs in anastrozole-resistant BC cells. Among these miRNAs, a few are crucial for AI-resistance: the overexpression of miR-125b or miR-205, or the silencing of miR-424 expression was sufficient to confer resistance to letrozole and anastrozole to sensitive BC cell lines, as a result of Akt/mTOR pathway activation [53]. Likewise, in a cohort of 65 BC patients, it was observed that the over-expression of miR-125b was associated with a poor prognosis and with relapse in ER-positive BC patients treated with endocrine therapy for the first time.

Remarkably, it was observed that the prognostic value of the miR-125 expression level was more informative than that of conventional biomarkers (ER, PR, or HER2), suggesting that this miRNA has a potential to be a tailored biomarker of poor prognosis and a strong candidate for therapies seeking to overcome letrozole resistance. KEGG (Kyoto Encyclopedia of Genes and Genomes) pathway analysis predicted that the dysregulation of miR-125b is sufficient to activate PI3K/Akt/mTOR [53].

In another study, it was demonstrated—using NanoString technology—that the expression level of miR-125b in cluster with let-7c and miR99a was decreased during the progression to endocrine resistance in luminal B BC; moreover, it was observed that miR-125b and let-7c regulate HER2 expression. These findings were correlated with data from samples profiled in The TCGA (Cancer Genome Atlas), and it was noted that the let-7c and miR-99a expression level was decreased in luminal B compared with luminal A tumors and only a tendency toward reduced miR-125b expression was observed in these same subsets. Thus, the authors suggested the use of this cluster as a biomarker of a poor outcome in ER positive BC patients [54].

There are only a few studies that describe miRNAs associated with Fulvestran resistance. Yu et al. reported that in a BC cell line resistant to this antiestrogen (antiestrogen-resistant MCF7/LCC9 cells), the overexpression of miR-214 sensitizes BC cells to Fulvestran through the inhibition of autophagy, by targeting UCP2 (uncoupling protein 2). Moreover, in 20 BC specimens, it was demonstrated that miR-214 was negatively correlated with UCP2. Thereby, the overexpression of UCP2 was correlated with Fulvestran resistance [55]. Another study demonstrated in vitro that miR-221/miR-222 confers resistance to Fulvestran; the authors identified multiple signaling pathways deregulated by these miRNAs, including p53, TGF-β, MAPK, Notch, ErbB, and Jak-STAT. The main targets of miR-221/222 were p27 and p57 (cell cycle inhibitors), whose downregulation modulates the effects of TGF-β and promotes ERα independent proliferation and tumor progression. This suggested that targeting these two miRNAs might be a potential therapeutic strategy for preventing the development of Fulvestran resistance [56]. The silencing of miR-21 was reported to confer sensitivity to Fulvestran and tamoxifen in BC cell lines (MCF-7) through enhancing the inhibition of the PI3K-Akt-mTOR pathway by directly targeting PTEN, increasing cell death through both apoptosis and autophagy. These results represent new knowledge about the therapeutic potential for ER positive BC patients [57].

### 2.6. miRNAs in SERM Therapy

SERMs are a group of drugs that bind ER in a tissue-specific fashion, counteracting their downstream signaling pathways and thus controlling cell proliferation [58]. The most prescribed SERM, Tamoxifen, is recommended by the ASCO (American Society of Clinical Oncology) guidelines for postmenopausal ER positive BC and for premenopausal BC patients for up to 10 years; it has been the most used treatment option in the past three decades [48]. Unfortunately, the effectiveness of this treatment is limited: around 40% of patients that receive it as adjuvant therapy manifested relapse [59]. As this drug is frequently used, more research groups are looking for miRNAs that underlie the resistance to it.

It has been reported that the expression of three miRNAS—miR-30a-3p, miR-30c, and miR-182—was associated with a better response to tamoxifen and a longer progression free survival (*p* < 0.01) in 246 ER positive advanced BC patients; however, only miR-30c was an independent predictor (*p* < 0.01), and a global pathway test predicted relation between miR-30c and both HER and RAC1 (Ras-related C3 botulinum toxin substrate 1 precursor) signaling pathways [60].

Hoppe et al., by means of a global miRNA screening from 1105 primary tumors of ER positive BC patients, provided data about the dysregulation of 20 miRNAs (eight downregulated and 12 overregulated); among others, miR-10a and miR-126 were predictive biomarkers of tamoxifen response in early BC disease, since they were associated with a longer relapse-free time, and the authors noticed that these miRNAs are linked with migration, invasion, and metastasis (*p* < 0.0001) [61].

These broad results must still be complemented through experimental and functional validation; a really hard task due to the sheer amount of information. Some groups have combined both approaches, such as, Rhothé et al., who analyzed a global miRNA expression profile from 89 ER positive BC patients and detected that miR-210 is clinically relevant due to its high expression being correlated with relapse-free survival (*p* = 0.004). Moreover, in vitro analysis performed in MCF7 cells demonstrated that this miRNA was involved in cellular events such as invasion, proliferation, and migration [62].

Several other reports analyzed single miRNAs and their role in tamoxifen resistance. Cittelly et al. showed that the downregulation of miR-342 was observed in multiple cell models derived from MCF-7; they were sensitized to tamoxifen-induced apoptosis upon restoring miR-342 expression, at which point they also displayed a dramatic reduction in cell growth. Functional studies revealed that miR-342 regulates genes involved with tumor cell death cancer pathways such as *GEMIN4* (Gem nuclear organelle associated protein 4) and *BMP7* (Bone morphogenetic protein 7). Besides, miR-342 was found to be underexpressed in a panel of tamoxifen refractory 20 BC tumors, which was related to recurrence or metastasis. Together, this data indicates that this miRNA is an important mediator of tamoxifen response and a potential candidate of miRNA therapy [51].

miR-10b overexpression was observed in tamoxifen-resistant cells, MCF7 BC cells (MCF7TR), and it was demonstrated that such resistance can be induced or reduced in MCF7 and T47D cells through miR-10b expression or silencing, respectively. In the same report, it was shown, through a luciferase assay, that HDAC4 (histone deacetylase) was a novel target of this miRNA; thus, HDAC4 is an important miR-10 target whose downregulation leads to tamoxifen resistance [63]. Another miRNA overexpressed in BC tamoxifen-resistant cells is miR-155, which targets SOC6 (suppressor of cytokine signaling 6). This interaction stimulates the STAT3 signaling pathway, leading to cell survival and resistance [64].

It has been reported that a consequence of tamoxifen treatment is the overexpression of the 14-3-3ζ protein and that its high expression is correlated with early disease recurrence. In vitro analysis showed that 14-3-3ζ protein downregulates miR-451 which, at the same time, inhibits 14-3-3ζ. Remarkably, the effect of miR-451 was specific of tamoxifen, but not of other SERMs such as fulvestran or raloxifene, and this could constitute a new mechanism of resistance to tamoxifen [23].

### 2.7. miRNAs in Targeted Therapies

#### 2.7.1. Trastuzumab

The human epidermal growth factor receptor-2 (HER2) is a cell surface receptor tyrosine kinase that is markedly amplified or over-expressed in approximately 20–30% of BC tumors and serves as a prognostic and predictive biomarker, as it is mainly is associated with a poor clinical outcome. HER2 enables the constitutive activation of growth factor signaling pathways leading to cell proliferation, survival, and tumorigenesis [65].

The combination of conventional chemotherapy with trastuzumab (or Herceptin)—a humanized monoclonal antibody that binds to domain IV of the extracellular segment of HER2—has proven successful in improving the overall survival of metastatic BC patients; nevertheless, about 50–60% of them do not respond to them, presenting with a lower clinical benefit and lower index of disease-free survival due to mechanisms of drug resistance [66].

Several mechanisms of HER2-inhibition resistance have been reported. The activation of epithelial to mesenchymal transition (EMT) has a preponderant role; it is a central biological event that contributes to tumor progression. It has been reported that HER2 overexpression regulates EMT through PI3K signaling activation [67]. Likewise, it was identified that the expression of miR-21 is higher in HER2-positive BC patients (*n* = 22). In this regard, Mattos-Arruda et al. demonstrated that miR-21 has an important role through the epigenetic silencing of PTEN, which is a negative regulator of PI3K. Therefore, the authors reported that miR-21 expression could be a potential biomarker for selecting trastuzumab-chemotherapy-resistant HER2 positive BC patients [68]. Another study reported, for the first time, a novel prognostic model based on the expression of two miRNAs (miR-150-5p and miR-4734) to improve the prediction of disease recurrence (seven relapsed versus seven non relapsed tumor tissues) [69].

miR-210 is another miRNA involved with the resistance to trastuzumab, and Jung et al. demonstrated that circulating levels of this miRNA were significantly higher in patients who had residual disease than in those who had a pathological complete response (pathological complete response *n* = 18 versus residual disease *n* = 11, *p* = 0.0359), so it is associated with a poor prognosis [70]. miR-210 targets the transcription factor E2F3 and the DNA repair enzyme RAD52, so might be the way in which it promotes cancer cell survival [71]. However, this function is not well known, and further functional studies are still needed to clarify this role.

On the other hand, in HER2 overexpression BC cell models (MCF7 and SKRB3), it was reported that IGF1R (insulin-like growth factor-1 receptor) has an important role during the development of resistance to trastuzumab [72]. In that regard, Ye et al. found that IGF1R is a direct target of miR-375 so that low levels of miR-375 were associated with resistance to this drug [73]. Epigenetic mechanisms such as DNA methylation and histone deacetylation were found to be responsible for the repression of miR-375 and therefore for the overexpression of IGF1R in trastuzumab resistant BC cells [73]. This finding suggested that miR-375 has a therapeutic potential for HER2 positive BC.

#### 2.7.2. Lapatinib

Lapatinib is a synthetic, orally-active tyrosine kinase inhibitor (TKI) that reversely blocks the phosphorylation of HER2, EGFR kinases, and p95HER2 (a trastuzumab resistance marker) and, consequently, there is a reduction of phosphorylation downstream of Akt and MAPK, which inhibits cell growth [74,75]. Currently, it is approved by FDA (Food and Drug Administration) for use in combination with capecitabine in HER2-positive BC metastatic patients that have received prior therapy with trastuzumab. Likewise, it is approved for use in combination with letrozole for postmenopausal HER2-positive metastatic patients [74,76].

It has been reported that the dual scheme of trastuzumab plus lapatinib improved the rates of pathological complete response and had the best outcome (three-year event free survival 86%, 95% confidence interval 75–92) in HER2-positive early BC patients [77,78]. Likewise, in combination with paclitaxel as first line treatment, lapatinib showed a better rate of overall survival compared with placebo plus paclitaxel in metastatic HER2 positive BC patients (20.5 vs. 27.8 months HR 0.74) [79]. However, these therapies do not produce a favorable outcome for every patient; and even those patients who respond initially will eventually show resistance [80].

The response to trastuzumab plus lapatinib treatment elicits miR-16 upregulation: which blocks extracellular PI3K/Akt signaling pathways. It was found that miR-16 inhibited cell proliferation in BC cell lines and in tumor samples resistant to these drugs; this suggested a tumor suppressor role for miR-16, as it targets CCNJ (Cyclin J) and FUBP1 (Far Upstream Element Binding Protein 1). This role was confirmed when high levels of miR-16 and low levels of CCNJ or FUBP1 correlated with trastuzumab and lapatinib sensitivity in preclinical models [81].

In another study, it was shown that miR-630 was significantly decreased in an HER2 positive BC tumor compared to matched peritumor tissue; interestingly, the authors observed that its inhibition in BC cell lines was associated with increased motility, migration, and invasion, and caused a significant increase in the resistance of lapatinib being its target of IGF1R, so they concluded that miR-630 could be a diagnostic and predictive biomarker for resistance to lapatinib and its therapeutic addition may enhance and improve patients’ response to this drug [82].

The over-expression of EGFR has been observed in more than 80% of all cases of TNBC (triple negative BC), suggesting that these tumors exhibit a sensitivity to EGFR-targeted agents [83]. In this regard, lapatinib seems to be a good treatment option for this cancer subtype, due to its anti-EGFR activity. In this sense, a randomized trial of paclitaxel with lapatinib versus placebo as first line treatment in metastatic BC noticed that paclitaxel with lapatinib has not shown an overall efficacy in TNBC, suggesting that EGFR tyrosine kinase activity may not be the major vulnerability in this subtype [84]. This can be explained by the findings reported by Hartman et al. They demonstrated the downregulation of miR-7 in triple negative BC cells, and, consequently, an increase of RAF-1/MAPK signaling pathway activation; this increases the expression and binding of c-jun to IL-6 (interleukine-6) promoter, which leads to IL-6 expression and enhanced migration in TNBC cells, constituting a plausible drug resistance mechanism [85].

### 2.8. Other Agents

Other drugs used as systemic therapy in BC are anthracyclines and cisplatin. There are few reports that describe miRNAs associated with the response to these therapies in BC; nonetheless, we approached the most recent findings. It is important to mention that the majority of these findings lack biological studies.

#### 2.8.1. Anthracyclines

Anthracyclines—such as doxorubicin, epirubicin, and Adriamycin—are agents used as adjuvant and neoadjuvant therapies. They interact with DNA base pairs and inhibit macromolecular biosynthesis; likewise, they are related with topoisomerase II after it has broken the DNA chain, stopping DNA replication. Their most important side effect is cardiotoxicity, which considerably hinders the scope of their use [86]. There are many studies that have evaluated the efficacy of antracyclines, but the most recent is a PELICAN trial: a phase III study that noticed that both doxorrubicin and capecitabine are effective as first line therapy for metastatic BC [87]. Another study reported that doxorrubicin is active in TNBC patients compared with non TNBC patients, while TNBC is associated with a bad prognosis, and some patients responded well, revealing molecular heterogeneity [88]. However, there are a few reports that describe miRNAs associated with the response to these drugs in BC. Nonetheless, we approached the most recent findings, although the greater part of them lack biological studies.

A recent preclinical study has reported miRNAs associated with doxorubicin: Lv J and collaborators noticed that miR-760 was significantly downregulated in doxorubicin-resistant BC cells and, through microarray analysis, they found 262 potential target genes of miR-760, which are involved in the cell cycle and TGF-β signaling pathway, with RHOB (Ras Homolog Family Member B), ANGOTL4 (Angiopoietin-like 4), and ABCA1 (ATP Binding Cassette Subfamily a Member 1) being the potential predicted targets of this miRNA. With these findings the authors suggested miR-760 as a prognostic biomarker during clinical treatment [89]. Likewise, another report showed 10 dysregulated miRNAs, three of which were upregulated: miR-141, miR-200c, and miR-31; and seven of which were downregulated: let-7e, miR-576-3p, miR-125b-1, miR-145, miR-765, and miR-760 in both BC cells and chemoresistant tissue. Thus, the authors propose them as biomarkers of prognosis of BC resistance [90], although further analysis is necessary to denominate them as biomarkers.

An miRNA expression signature was obtained from 15 TNBC patients that received anthracyclines or taxanes-based adjuvant chemotherapy, including upregulated miRNAs (miR-155-5p, miR-21-3p, miR-181a-5p, miR-181b-5p, and miR-183-5p) and six downregulated miRNAs (miR-10b-5p, miR-451a, miR-125b-5p, miR-31-5p, miR-195-5p, and miR-130a-3p). From them, it was demonstrated in vitro that miR-130a-3p and miR-451 significantly changed the sensitivity to doxorubicin in the BC cell line MDA-MB-231; with these results, the authors suggested that the abnormal expression of miRNAs is part of the reason why they are chemoresistant [91]. This finding is supported by Kovalchuk and collaborators, who reported that an enforced increase of miR-451 in cell lines doxorubicin-resistant increases its sensitivity through the downregulation of the *MDR1* (multidrug resistance 1) gene, so that this interaction has an important consequence in the sensitivity of doxorubicin [92].

In order to identify an miRNA signature as a biomarker capable of predicting the clinical response to taxane-anthracycline-based neoadjuvant chemotherapy, a microarray from 21 BC patients was performed, and it was observed that an elevated expression of miR-125b and miR-141 was associated with chemoresistance. This data was confirmed in BC cell lines, in which the overexpression of these miRNAs markedly inhibited taxane-anthracycline-induced cell cytotoxicity. Pathway analysis demonstrated that miR-125b and miR-141 are involved in cell cycle control and apoptotic pathways, although the authors did not describe a specific target in either biological process [93]. miR-181a is another miRNA associated with poor disease survival and overall survival for TNBC patients treated with doxorubicin. Biological assays in TNBC cell lines demonstrated that this miRNA was upregulated by NF-κB-facilitated IL-6 induction—a signaling pathway activated after doxorubicin treatment—thus, miR-181a suppression increased the response and reduced lung metastasis in the orthotropic model due to *Bax* (BCL2 associated X, Apoptosis Regulator) gene targeting, which suggested that miR-181a is a potential therapeutic strategy [94].

In the same way, Pierluigi and collaborators identified a signature of four miRNAs: miR-155, miR-493, miR-30e, and miR-27a, which discriminate between patients with a low and high risk of prognosis. The authors suggested that other treatment options should be considered for patients that express this miRNA signature [95]. On the other hand, in a clinical study, the serum of 68 patients that received epirubicin and paclitaxel as a neoadyuvant chemotherapy was analyzed and the overexpression of miR-19a and miR-205 was found in the serum of resistant patients, which may be useful to predict chemosensitivity in BC patients [96]. miR-222 is an miRNA associated with adriamycin through PTEN, the Akt/FOXP1 pathway, and its high expression is associated with overall survival by TCGA data [97].

#### 2.8.2. Cisplatin

Cisplatin is a DNA cross-linking agent that blocks DNA replication and transcription in order to induce apoptosis of the tumor cells. This chemotherapeutic agent is not used routinely for BC; however, preclinical data suggests that TNBC breast cancers are more sensitive to this agent due to deficiencies in the BRCA-associated DNA repair mechanism producing higher rates of response for the subset of the BRCA mutation versus non-BRCA mutation. This data suggests that the heterogeneity among patients is the basis of their diverse responses to cisplatin: likewise, repetitive and long-term administration of cisplatin induces resistance [98].

Some studies have indicated that the curative effect of cisplatin can be increased or inhibited through targeting apoptosis and cell migration. For instance, through microarrays analysis, 46 overexpressed and 57 underexpressed miRNAs out of 103 were identified in cisplatin-resistant MCF-7 cells. The most significantly altered miRNAs were miR-146a, miR-10a, miR-221/222, miR-345, miR200b, miR-200c, and miR-345 targeting MRP1, and this result confirms that the dysregulation of miRNA expression is associated with drug resistance mechanisms [99].

It was reported that miR-221 was significantly overexpressed in BC cell lines and in 35 BC specimens; thus, the knockdown of miR-221 promotes the cytotoxicity of cisplatin in BC cells since this miRNA targets the BIM-Bax/Bak axis. These findings emphasize the role of this axis in cisplatin treatment to BC [100]. Likewise, it was noticed that miR-944 plays an important role in cell proliferation, migration, and invasion. This miRNA was found to be overexpressed in BC cisplatin-resistant cells and targets the *Bnip3* gene, a proapoptotic Bcl2-family member that belongs to the BH3-only subfamily of Bcl-2 family proteins. Therefore, a knockdown of miR-944 re-sensitized the cisplatin-resistant BC cells [101].

In another study, it was explored in vitro and in pre-clinical models that miR-519d increases the sensitivity to cisplatin by targeting MCL-1 (Myeloid cell leukemia 1)—an important anti-apoptotic protein in cancer—in BC stem cells [102]. Another miRNA involved in the response to cisplatin is miR-199a-3p, which is expressed at low levels in BC cells lines resistant to cisplatin; but when overexpressed, it increased the sensitivity to cisplatin thorough targeting TFAM (mitochondrial transcription factor A), an important protein for maintaining mitochondrial biogenesis. Then, the authors noticed that miR-199a-3p promoted cisplatin-induced apoptosis, as well as an anti-proliferative effect in the MDA-MB-321 cell line, attenuating cisplatin resistance [103]. miR-302b overexpression improved the sensitivity to cisplatin in BC cell lines, since it regulates the E2f1-ATM axis, targeting E2F1 directly. Thus it decreases cellular viability and proliferation in different BC cell lines; the authors suggested that miR-302b might represent a biomarker that predicts the response to cisplatin treatment [104].

A clinical study reported that high levels of miR-218 were associated with a significantly increased survival of 85 BC patients treated with cisplatin and, interestingly, this miRNA inhibits BRCA1 expression. However, the authors suggested further studies using preclinical animal models to confirm this data [105]. Another miRNA that directly regulates BRCA1 expression in BC is miR-638. This miRNA exerts distinct effects on cell proliferation and invasion, enhancing chemotherapy sensitivity in TNBC cells, which suggests that this miRNA may serve as a potential prognostic biomarker [106].

#### 2.8.3. Capecitabine, Gemcitabine, and Vinorelbine

Consistent with the NCCN (National Comprehensive Cancer Network) guidelines, other single agents included in the systemic therapy that is mainly administered to metastatic BC patients are capecitabine, gemcitabine, and vinorelvine. There are few studies that report miRNAs as biomarkers of response to these chemotherapy drugs.

Capecitabine is a prodrug that is converted to fluorouracil by the enzyme thymidine phosphorylase. A phase trial analyzed patients that have progressed on anthracyclines and that received capecitabine versus paclitaxel; both drugs were similar in efficacy and the overall response rate (36% for capecitabine and 26% for paclitaxel) [107]. Regarding miRNAs as predictors of response to this drug, it was reported that the miRNA profile of BC patients treated with capecitabine in combination with ixabepilone showed the deregulation of miR-122a, miR-145, and miR-205, which were found be significantly under-expressed in luminal androgen receptor-type TNBC compared with normal tissues. Despite the lack of biological assays to determine the function of these miRNAs, the authors suggested that these findings provide the basis for future clinical trials [108].

Vinorelbine is an alkaloid that obstructs microtubule assembly and blocks the formation of the metaphasic mitotic spindle, preventing cell division; it is one of the most active cytotoxic agents in BC—especially to metastatic BC—and causes less toxicity compared to other chemotherapy agents [109]. In a phase III trial, the overall response rate was similar for both vinorelbine alone and in combination with gemcitabine (28.4% vs. 24.3%) (21937705). Zhong and collaborators reported, through the microarray analysis of BC cell line-vinorelvine-resistant (MDA-MB-231), 11 upregulated mi-RNAs: miR-138-5p, miR-182-5p, miR-18a-5p, miR-193b-3p, miR-199a-5p, miR-210-3p, miR-21-5p, miR-378a-3p, miR-4262, miR-4725-5p, and miR-92b-3p; and six downregulated miRNAs: let-7a-5p, miR-130a-3p, miR-146a-5p, miR-221-3p, miR-23b-3p, and miR-4319. The signaling pathways affected by these miRNAs were MAPK, mTOR, Wnt, and TGF-β, and these findings provided a global view about the function of differential expression miRNAs related to drug resistance in breast cancer [110].

Gemcitabine (2′,2-difluorodeoxycytidine) is a nucleoside analogue which requires intracellular phosphorylation to produce the nucleotides gemcitabine diphosphate (dFdCDP) and triphosphate (dFdCTP), which are then incorporated into DNA and RNA [111]. A phase III trial noticed that the addition of gemcitabine to paclitaxel does improve the complete response in neoadjuvant chemotherapy [112]. In BC, it is an effective drug for metastatic disease, but around 30% of patients did not response this drug [111]. In this regard, it was described that miR-21 overexpression mediates resistance to gemcitabine. Interestingly, gemcitabine-resistant BC cells exhibit enhanced properties of EMT. They acquire a more aggressive mesenchymal phenotype with more motile and invasive characteristics through targeting PTEN and, consequently, activating the Akt pathway. With these findings, the authors suggested that miR-21 is a promising predictor of gemcitabine resistance [113].

## 3. Conclusions

**Figure 1:**
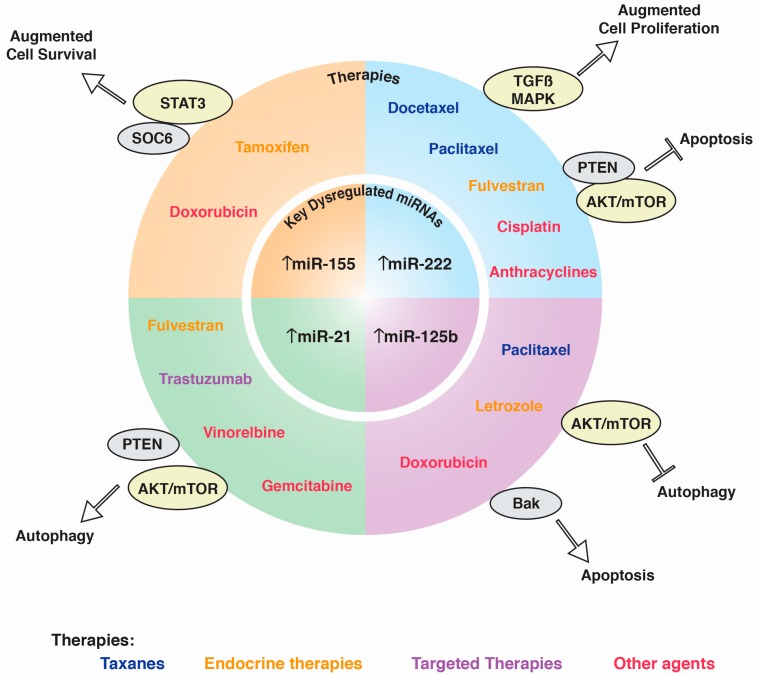
Key miRNAs in the resistance to BC treatment. Upregulation of four key miRNAs—miR-155, miR-222, miR-125b, and miR-21—is associated with the resistance to systemic therapy: Taxanes, Endocrine therapies, Targeted therapies, and Other agents. When dysregulated, these miRNAs disrupt genes or pathways (denoted by gray or yellow ovals, respectively) that lead to the molecular mechanisms associated with four of the hallmarks of cancer. Up-arrows denote up-regulated miRNA whereas T-Bar denote stop signaling pathway.

Resistance to BC treatments is still a global health issue, so many efforts are now directed toward finding potential predictors of the response to treatments and therapeutic alternatives; notably, miRNAs can play both roles. We have reviewed the literature on miRNAs that mediate the resistance or predict sensitivity to the current systemic BC treatments and found controversial results in some instances. To date, most studies in this area are still preclinical and thus lack conclusive results from patient cohorts; this strongly suggests that there is still a long road ahead towards the identification of miRNAs as bona-fide indicators of treatment success or failure, but, at the same time, shows that we are on the correct path, as the results are promising. The evidence that we reviewed here has let us identify four miRNAs that mediate resistance to multiple therapeutic strategies when overexpressed. According to the published literature, these key miRNAs target genes and pathways with important implications in cancer development. In Figure 1, we show how these targets are closely related with cellular events previously identified as hallmarks of cancer. We think that the fact that a few dysregulated miRNAs elicit cellular responses of such importance is not a coincidence, and these miRNAs are strong candidates for further research such as functional studies and clinical trials since the mechanisms that they form a part of are still partially unknown, but deeply interesting in order to use them in the future in the clinical practice as predictors of the response to systemic treatments. This information would provide a comprehensive molecular background of the response to each treatment, so that more informed decisions can be taken in personalized therapy, leading to an improved quality of life and extended survival. Besides, the quest for miRNAs with a response predictor nature is far for complete, as seen when comparing the small fraction of defined miRNAs that have been analyzed in the presented works with the total number of described miRNAs. We expect that future reviews like this one will cover a much wider amount of research and offer even more tantalizing conclusions.

## Figures and Tables

**Table 1:** miRNAs as potential biomarkers of resistance to treatments in breast cancer.

Overexpressed miRNA	Predicted Targets	Pathway Involved	Reference
Paclitaxel			
miR-Lin28	Let7a	miRNAs processing	[20]
miR-125b	Bak1	Apoptosis	[21]
miR-520h	DAPK2	PI3K/Akt signaling	[22]
miR18a		mTOR signaling	[25]
Docetaxel			
miR-129-3p	CP110	G2/M progression and apoptosis	[31]
miR-141	EIF4E	Apoptosis	[30]
miR-3646	GSK-3β	β-catenin signaling pathway	[32]
miR-452	APC4		[33]
miR-663	HSPG2		[34]
miR-34a	BCL-2 and CCND1	Apoptosis	[35]
miR-222 and miR-29a	PTEN	Apoptosis	[39]
Letrozol + anastrozole			
miR-125b and miR-205		Akt/mTOR pathway	[53]
Fulvestrant			
miR-221 and miR-222	AXIN2, SFRP2, CHD8 and NLK	p53, TGF-β, MAPK, Notch, ErbB and Jak-STAT	[56]
miR-21	PTEN	PI3K-Akt-mTOR pathway	[57]
Tamoxifen			
miR-10b	HDAC4	Epithelial-mesenchymal transition	[63]
miR-210	RAD52	Invasion, proliferation and migration	[70]
mirR-155	SOC6	STAT3 signaling pathway	[62]
Trastuzumab			
miR-21	PTEN	PI3K-Akt-mTOR/epithelial-to-mesenchymal transition and inflammatory signals	[67]
miR-150-5p and miR-4734			[68]
miR-210	RAD52	Cell survival	[69]
Doxorrubicin miR-141 miR-200c miR-31		MAPK signaling pathway, regulation of the actin cytoskeleton, cytokine-cytokine receptor interaction	[90]
miR-155p miR-21-3p miR-181a-5p miR-181b-5p miR-183-5p		PTEN/Akt, MAPK, MDR1, RhoA, FOXO3 and PDCD4 pathway	[91]
miR-125b miR-141		cell cycle control and apoptotic pathways	[93]
miR-155 miR-493 miR-30e miR-27a			[95]
miR-181a	Bax		[94]
Epirubicin miR-19a miR-205	PTEN		[96]
Adriamycin miR-222	PTEN	PTEN, Akt/FOXP1 pathway	[97]
Cisplatin miR-146a miR-10a miR-221/222 miR-345 miR200b and miR-200c	MRP1	EMT efflux of drugs	[99]
miR-221		BIM-Bax/Bak axis	[100]
miR-944	BNIP3	cell proliferation, migration and invasion	[101]
Gemcitabine miR-21	PTEN	EMT	[113]
Vinorelbine miR-138-5p, miR-182-5p, miR-18a-5p, miR-193b-3p, miR-199a-5p, miR-210-3p, miR-21-5p, miR-378a-3p, miR-4262, miR-4725-5p and miR-92b-3p		MAPK, mTOR, Wnt, and TGF-β	[110]

**Table 2:** miRNAs as potential biomarkers of sensitivity to treatments in breast cancer.

Overexpressed miRNA	Predicted Targets	Pathway Involved	Reference
Paclitaxel			
miR-451	Bcl-2	Apoptosis	[23]
miR-100	mTOR	Cell proliferation and survival	[24]
Docetaxel			
miR-139-5p	Notch1	Cell growth and apoptosis	[36]
miR-205		cell proliferation and clonogenic potential	[37]
miR-125a-3p	BRCA1		[38]
Fulvestran			
miR-214	UCP2	Autophagy	[55]
Tamoxifen			
miR-30c		HER2 and RAC1 signaling pathway	[59]
MiR-10a and miR-126			[60]
Lapatinib			
miR-16	CCNJ and FUBP1	PI3K/Akt signaling pathways	[80]
Cisplatin miR-519d	MCL-1	anti-apoptotic signaling pathway	[102]
miR-199a-3p	TFAM	mitochondrial biogenesis	[103]
miR-302b	E2F1	E2f1-ATM axis	[104]
miR-218, miR-638	BRCA1	DNA reparation, cell proliferation and invasion	[105,106]

## Abbreviations

BC	Breast Cancer
miRNA	MicroRNA
Bak1	Pro-apoptotic Bcl2 antagonist killer 1
DAPK2	Death-Associated kinase 2
TSP-1	Thrombospondin-1
MCL-1	Myeloid cell leukemia
EIF4E	Eukaryotic translation initiation factor 4E
CP110	Centriolar coiled-coil protein 110
APC4	Anaphase promoting complex subunit 4
HSPG2	Heparin sulfate proteoglycan 2
CCND1	Cyclin D1
PTEN	Phosphatase and tensin homolog
ERα	Estrogen receptor α
SERDs	Selective estrogen receptor downregulators
SERMs	Selective ER modulators
TCGA	The cancer genome atlas
ASCO	American society of clinical oncology
GEMIN4	Gem nuclear organelle associated protein 4
BMP7	Bone morphogenetic protein 7
UCP2	Uncoupling protein 2
HDAC4	Histone deacetylase
SOC6	Suppressor of cytokine signaling 6
HER2	Human epidermal growth factor receptor-2
EMT	Epithelial to mesenchymal transition
IGF1R	Insulin-like growth factor-1 receptor
TKI	Tyrosine kinase inhibitor
FDA	Food and drug administration
CCNJ	Cyclin J
FUBP1	Far upstream element binding protein 1
TNBC	Triple negative breast cancer
IL-6	Interleukine-6
